# Reviewed article: Bando DH, Neto FC, Queiroz AP. Stroke mortality negatively associated with health, education, and safety: an ecological study, Minas Gerais, 2014-2022. Epidemiol Serv Saude. 2025:34;e20240820

**DOI:** 10.1590/S2237-96222025v34e20240820.c

**Published:** 2025-09-29

**Authors:** Andreza Maria Luzia Baldo de Souza

**Affiliations:** 1 Universidade Estadual de Campinas, Campinas, SP, Brazil

## Peer review C

## Questionnaire

Does the manuscript contain new and significant information to justify publication? Yes

Does the Abstract (Summary) clearly and accurately describe the content of the article? Yes

Is the problem significant and concisely stated? Yes

Are the methods described comprehensively? Yes

Are the interpretations and conclusions justified by the results? Yes

Is adequate reference made to other work in the field? Yes

## Manuscript Structure

Length of article is: Adequate

Number of tables is: Adequate

Number of figures is: Adequate

Please state any conflict(s) of interest that you have in relation to the review of this paper. None

Interest: Excellent

Quality: Good

Originality: Good

Overall: Good

Recommendation: Minor Revision

## Comments

Parabéns aos autores por abordar um tema tão relevante, agumas sugestões: Justificar melhor a escolha do período de estudo (2014-2022) e a utilização do dado sociodemográfico de 2018, que representa um único ponto no tempo.

Explicar com mais clareza como foi determinada a janela máxima para a detecção dos conglomerados espaço-temporais.

Discutir possíveis vieses na identificação das taxas de mortalidade, como variações na qualidade dos registros de óbitos entre municípios. O R² de 0,41 sugere uma boa capacidade explicativa do modelo, mas a interpretação dos coeficientes poderia ser discutida com mais profundidade.

A discussão está bem fundamentada, mas poderia explorar mais as implicações desses achados para a formulação de políticas públicas e estratégias de intervenção.

